# Comparisons of allometric and climate-derived estimates of tree coarse root carbon stocks in forests of the United States

**DOI:** 10.1186/s13021-015-0032-7

**Published:** 2015-09-04

**Authors:** Matthew B. Russell, Grant M. Domke, Christopher W. Woodall, Anthony W. D’Amato

**Affiliations:** 1 Department of Forest Resources, University of Minnesota, St. Paul, MN 55108 USA; 2 USDA Forest Service, Northern Research Station, St. Paul, MN 55108 USA; 3 Rubenstein School of Environment and Natural Resources, University of Vermont, Burlington, VT 05405 USA

**Keywords:** Belowground biomass, Carbon accounting, Carbon-climate, Root:shoot ratio, Forest Inventory and Analysis

## Abstract

**Background:**

Refined estimation of carbon (C) stocks within forest ecosystems is a critical component of efforts to reduce greenhouse gas emissions and mitigate the effects of projected climate change through forest C management. Specifically, belowground C stocks are currently estimated in the United States’ national greenhouse gas inventory (US NGHGI) using nationally consistent species- and diameter-specific equations applied to individual trees. Recent scientific evidence has pointed to the importance of climate as a driver of belowground C stocks. This study estimates belowground C using current methods applied in the US NGHGI and describes a new approach for merging both allometric models with climate-derived predictions of belowground C stocks.

**Results:**

Climate-adjusted predictions were variable depending on the region and forest type of interest, but represented an increase of 368.87 Tg of belowground C across the US, or a 6.4 % increase when compared to currently-implemented NGHGI estimates. Random forests regressions indicated that aboveground biomass, stand age, and stand origin (i.e., planted versus artificial regeneration) were useful predictors of belowground C stocks. Decreases in belowground C stocks were modeled after projecting mean annual temperatures at various locations throughout the US up to year 2090.

**Conclusions:**

By combining allometric equations with trends in temperature, we conclude that climate variables can be used to adjust the US NGHGI estimates of belowground C stocks. Such strategies can be used to determine the effects of future global change scenarios within a C accounting framework.

**Electronic supplementary material:**

The online version of this article (doi:10.1186/s13021-015-0032-7) contains supplementary material, which is available to authorized users.

## Background

The management of forest ecosystems and their associated carbon (C) stocks has become an important global strategy for reducing greenhouse gas (GHG) emissions and possibly mitigating future effects of climate change [1–3]. Societal demands and trends in land use, in combination with future global change scenarios, may reduce the amount of C stored in forests and associated wood products [4]. As a result, there are substantial knowledge gaps regarding the C implications of various forest management activities, which may arise from the complex pathways of C emissions and sequestration in forest ecosystems [2, 3]. In addition, the logistical and methodological constraints associated with estimating C in certain forest ecosystem components across large areas, namely belowground pools, has hampered the development of accurate estimates, creating a need for refined modeling approaches to quantify belowground C stocks.

From an ecological perspective, the use of plant characteristics such as root to shoot ratio and root mass fraction have aided investigators in understanding belowground C stocks associated with coarse roots. For example, Poorter et al. [5] presented a mean root mass fraction of 0.21 for temperate and boreal forests and Smyth et al. [6] calculated a root to shoot ratio of 0.256 across Canada. These proportions lend insight into partitioning effects and belowground C stores. Various studies have examined the degree to which these ratios are altered under various stand and environmental conditions. Litton et al. [7] found that partitioning to belowground components increased with stand density in lodgepole pine forests (*Pinus contorta* Dougl. ex Loud. var. *latifolia* Engelm.), which may in turn be related to tree size. Root mass fractions have been found to decrease with total plant biomass consistently among angiosperm and gymnosperm forests [5, 8]. In tropical systems, 15–20 year-old plantations allocated more C belowground when compared to mature broad-leaved forests [9], highlighting the importance of accounting for management scenarios in assessments of belowground C stores. However, application of these findings to forest C accounting activities has been limited as few studies measure all components of the C budget (e.g., biomass, flux, and partitioning; [7]).

The monitoring of belowground C has incorporated a number of these ecological insights using a variety of approaches at different scales. Allometric equations designed at the individual-tree level are common for determining belowground C [e.g., 10, 11]. Throughout the United States (US), belowground biomass of coarse roots is commonly estimated using the equations of Jenkins et al. [12] as a ratio of total aboveground biomass and tree diameter at breast height (DBH; [13]). Although allometric equations tend to account for a large portion of the apparent variability associated with belowground biomass (e.g., *R* ^2^ values range from 0.77 to 0.96; Litton et al. [11]), there are a few drawbacks to this approach. First, these equations tend to rely on DBH and are not explicitly constructed to estimate belowground C [14]. Secondly, allometric equations have not historically incorporated climate information that integrates differences in ecosystem productivity and allows for evaluations of future climate change scenarios on global C cycles. Highlighting this concern, Reich et al. [8] recently compiled a global dataset and concluded that forest biomass found in roots was inversely related to mean annual temperature, suggesting that climate may act as a driver of belowground C allocation.

Globally, there has been an increased interest in recent years for refining forest carbon estimation to understand greenhouse gas emissions in support of the United Nations Framework Convention on Climate Change [15, 16]. Forest C stocks in the US are estimated using data collected by the US Forest Service, Forest Inventory and Analysis (FIA) program. In the current national greenhouse gas inventory (NGHGI; [17]), belowground stocks are estimated in two stages by first quantifying total aboveground biomass using allometric equations then estimating a ratio of coarse root to total aboveground biomass [12, 13]. As observations of belowground tree biomass and C are often limited [14], relying on allometric equations has been necessary to obtain estimates from strategic-scale forest inventories such as FIA’s. At the same time, the lack of empirical information across a diverse array of tree species in temperate forests such as those found throughout North America encourages researchers to test alternative approaches for quantifying belowground biomass and C. Exploring belowground C modeling approaches that incorporate climatic attributes may both adjust our estimates of coarse root C stocks at national scales (i.e., application in the US NGHGI) while enhancing evaluations of future climate change scenarios on forest C cycles.

The overall objective of this research is to adjust belowground C estimation procedures for reporting in the US NGHGI. Specific objectives are to (1) estimate belowground C stocks by employing individual tree- and stand-level methodologies, (2) adjust estimates of belowground C stocks by combining allometric and climate-derived approaches using current and projected climate attributes, and (3) compare alternative estimation approaches for belowground C stocks for future application in the US NGHGI.

## Results

**Fig. 1:**
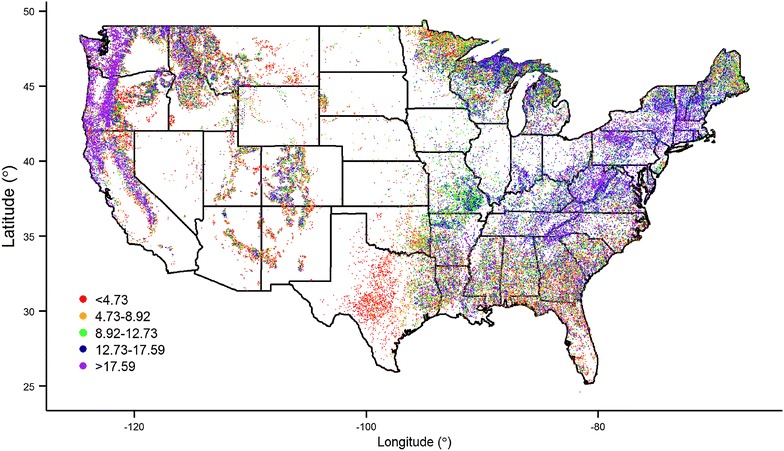
Distribution of live-tree belowground C estimates from the model of Reich et al. [8] (BGC_Clim_; Mg ha^−1^)

Estimates of belowground carbon (BGC) from approaches currently employed in the US NGHGI suggest that C stocks are dependent on geographic region and forest type. Mean values of belowground carbon in the US greenhouse gas inventory (BGC_NGHGI_) were small in short-statured, open forests such as pinyon-juniper and woodland hardwood types (typically less than 2 Mg ha^−1^). Mean BGC_NGHGI_ was largest in hemlock-Sitka spruce forests in the Pacific Northwest [40.76 ± 0.96 Mg ha^−1^ (mean ± SE)] and redwood forests in the Pacific Southwest (59.27 ± 7.06 Mg ha^−1^). For climate-derived estimates of belowground C, belowground carbon from climate-derived models (BGC_Clim_) stock estimates were slightly smaller in magnitude compared to BGC_NGHGI_ estimates [e.g., hemlock-Sitka spruce (33.82 ± 0.80 Mg ha^−1^) and redwood forests (45.64 ± 5.44 Mg ha^−1^)] and generally showed decreasing C at lower latitudes (Fig. 1). On average, BGC_Clim_ estimates were 0.60 Mg ha^−1^ greater than current BGC_NGHGI_ models when considering all forest types (Additional file 1: Table S1).

**Fig. 2:**
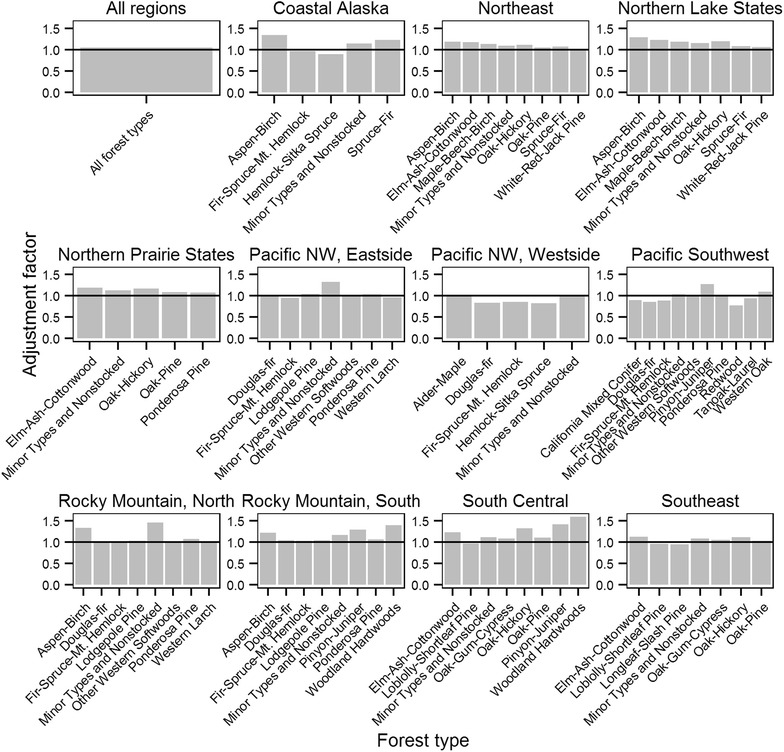
Adjustment factors used to amend live-tree belowground C estimates from the current US national greenhouse gas inventory (BGC_NGHGI_; Smith et al. [13]) with climate-derived predictions (BGC_Clim_; Reich et al. [8]) by US region and forest type. Adjustment factors less than and greater than one indicate less and more belowground C when climate-derived predictions are used. See Additional file 1: Table S1 for mean belowground C values and adjustment factors

**Fig. 3:**
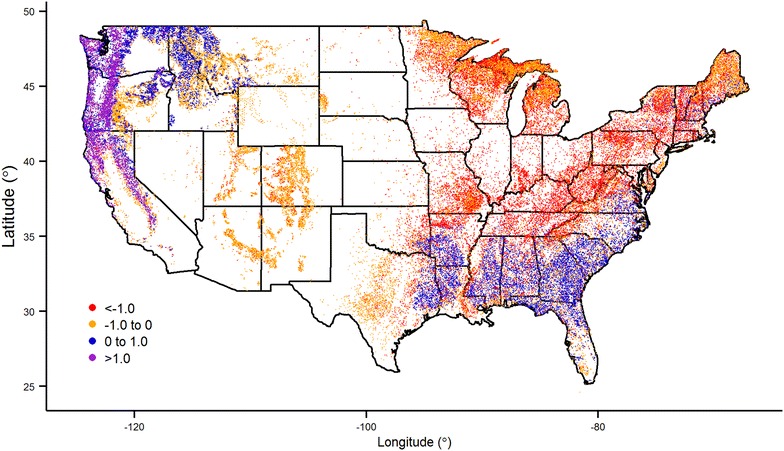
Distribution of differences between live-tree belowground C estimates and adjusted estimates (BGC_NGHGI_ − BGC_ClimAdj_; Mg ha^−1^), with *red colors* indicating higher estimated C and *purple colors* less C

The adjustment factors used to align climate-derived predictions of belowground C with the US NGHGI approach ranged from 0.77 to 1.60 with little variability within a region of interest (Fig. 2; Additional file 1: Table S1). Equivalence tests used to contrast the two approaches for estimating belowground C with a null hypothesis of dissimilarity and a threshold of ±25 % were rejected for 20 out of the 78 forest types examined. Equivalence tests were generally rejected for forest types that displayed relatively low and high BGC stocks, e.g., pinyon-juniper and woodland hardwoods (low C stocks) and redwood forest types (high C stocks). Mean differences were generally largest across the Pacific Northwest (Westside), indicating climate-adjusted predictions estimated less belowground C compared to allometric-derived estimates in this region. Generally, negative mean differences were observed across most forest types, indicating that climate-adjusted models predict greater belowground C stocks (e.g., mean percent difference was −5.2 % greater across all forest types; Table 1). Compared to current NGHGI models, model differences showed greater belowground C stocks occurring in the Appalachian Mountain region and areas where northern hardwood forests are common, e.g., in the upper Midwest and northeastern US states. Conversely, areas of smaller belowground C stocks were identified across the Pacific Northwest and Southeast US (Fig. 3). This was further reflected when population estimates were scaled to the state level. The states of Oregon and Washington were predicted to display the largest negative mean difference in belowground C stocks (−10.6 and −10.7 %, respectively). Conversely, the largest mean positive difference in belowground C stocks was in the states of Kentucky, Tennessee, and Oklahoma (28.0, 26.7, and 22.6 %, respectively). This represents a total estimated increase of 368.87 Tg of belowground C across the US, or a 6.4 % increase when compared to currently implemented NGHGI models (Table 2).

**Table 1:** Equivalence test results (critical threshold of ±25 %) comparing live-tree belowground C estimates from the current US national greenhouse gas inventory (BGC_NGHGI_) [13] to adjusted estimates (BGC_ClimAdj_)

Region	Forest type	*n*	BGC_NGHGI_ − BGC_ClimAdj_			
			Mean difference	Mean % difference	SE difference	Result^a^
All regions	All forest types	70,126	−0.58	−5.2	0.01	E
Coastal Alaska	Aspen-Birch	78	−1.71	−34.5	0.16	NE
Coastal Alaska	Fir-Spruce-Mt. Hemlock	342	0.41	3.4	0.02	E
Coastal Alaska	Hemlock-Sitka Spruce	571	2.75	10.6	0.07	E
Coastal Alaska	Minor types and nonstocked	85	−0.77	−13.8	0.13	NE
Coastal Alaska	Spruce-Fir	105	−0.60	−23.0	0.05	NE
Northeast	Aspen-Birch	367	−1.58	−18.2	0.05	E
Northeast	Elm-Ash-Cottonwood	205	−1.76	−16.9	0.08	NE
Northeast	Maple-Beech-Birch	3845	−1.78	−12.8	0.01	E
Northeast	Minor types and nonstocked	346	−0.97	−8.5	0.03	E
Northeast	Oak-Hickory	2509	−1.67	−11.1	0.01	E
Northeast	Oak-Pine	247	−0.68	−5.0	0.02	E
Northeast	Spruce-Fir	900	−0.59	−6.6	0.01	E
Northeast	White-Red-Jack Pine	380	0.00	−0.1	0.00	E
Northern Lake States	Aspen-Birch	2477	−1.82	−28.8	0.02	NE
Northern Lake States	Elm-Ash-Cottonwood	971	−1.91	−22.7	0.04	NE
Northern Lake States	Maple-Beech-Birch	2956	−2.08	−18.1	0.02	E
Northern Lake States	Minor types and nonstocked	460	−0.94	−15.2	0.04	E
Northern Lake States	Oak-Hickory	1845	−2.05	−20.0	0.03	E
Northern Lake States	Spruce-Fir	1854	−0.51	−8.4	0.01	E
Northern Lake States	White-Red-Jack Pine	935	−0.52	−5.5	0.01	E
Northern Prairie States	Elm-Ash-Cottonwood	342	−1.88	−19.0	0.06	NE
Northern Prairie States	Minor types and nonstocked	465	−1.26	−13.0	0.04	E
Northern Prairie States	Oak-Hickory	3265	−1.80	−17.2	0.01	E
Northern Prairie States	Oak-Pine	207	−0.76	−9.0	0.03	E
Northern Prairie States	Ponderosa Pine	164	−0.54	−7.6	0.03	E
Pacific Northwest, Eastside	Douglas-fir	992	0.27	2.3	0.01	E
Pacific Northwest, Eastside	Fir-Spruce-Mt. Hemlock	948	0.82	4.5	0.02	E
Pacific Northwest, Eastside	Lodgepole Pine	571	−0.37	−4.6	0.01	E
Pacific Northwest, Eastside	Minor types and nonstocked	213	−0.91	−33.1	0.09	NE
Pacific Northwest, Eastside	Other Western Softwoods	505	−0.06	−0.5	0.00	E
Pacific Northwest, Eastside	Ponderosa Pine	1446	−0.36	−4.2	0.01	E
Pacific Northwest, Eastside	Western Larch	136	0.65	4.3	0.03	E
Pacific Northwest, Westside	Alder-Maple	226	0.18	0.5	0.01	E
Pacific Northwest, Westside	Douglas-fir	2130	5.79	16.3	0.09	E
Pacific Northwest, Westside	Fir-Spruce-Mt. Hemlock	553	4.27	13.6	0.13	E
Pacific Northwest, Westside	Hemlock-Sitka Spruce	552	6.93	16.7	0.16	E
Pacific Northwest, Westside	Minor types and nonstocked	355	0.28	1.8	0.01	E
Pacific Southwest	California Mixed Conifer	946	2.30	9.9	0.05	E
Pacific Southwest	Douglas-fir	139	4.45	14.1	0.27	E
Pacific Southwest	Fir-Spruce-Mt. Hemlock	243	2.81	10.7	0.12	E
Pacific Southwest	Minor types and nonstocked	116	−0.25	−2.8	0.04	NE
Pacific Southwest	Other Western Softwoods	382	−0.16	−2.3	0.01	E
Pacific Southwest	Pinyon-Juniper	31	−0.57	−26.7	0.12	NE
Pacific Southwest	Ponderosa Pine	242	0.00	0.0	0.00	E
Pacific Southwest	Redwood	64	13.63	22.6	1.62	NE
Pacific Southwest	Tanoak-Laurel	207	1.51	5.6	0.07	E
Pacific Southwest	Western Oak	778	−0.98	−9.9	0.03	E
Rocky Mountain, North	Aspen-Birch	98	−1.46	−32.7	0.15	NE
Rocky Mountain, North	Douglas-fir	1659	−0.11	−1.4	0.00	E
Rocky Mountain, North	Fir-Spruce-Mt. Hemlock	1467	0.00	0.3	0.00	E
Rocky Mountain, North	Lodgepole Pine	828	−0.30	−2.6	0.01	E
Rocky Mountain, North	Minor types and nonstocked	331	−0.70	−45.8	0.06	NE
Rocky Mountain, North	Other Western Softwoods	329	0.23	2.1	0.01	E
Rocky Mountain, North	Ponderosa Pine	499	−0.47	−6.6	0.02	E
Rocky Mountain, North	Western Larch	128	0.26	1.7	0.02	E
Rocky Mountain, South	Aspen-Birch	647	−1.77	−22.4	0.05	NE
Rocky Mountain, South	Douglas-fir	420	−0.41	−3.5	0.01	E
Rocky Mountain, South	Fir-Spruce-Mt. Hemlock	1061	−0.34	−3.4	0.01	E
Rocky Mountain, South	Lodgepole Pine	359	−0.41	−4.0	0.01	E
Rocky Mountain, South	Minor types and nonstocked	166	−0.76	−16.7	0.07	NE
Rocky Mountain, South	Pinyon-Juniper	647	−0.41	−28.9	0.02	NE
Rocky Mountain, South	Ponderosa Pine	924	−0.45	−6.1	0.01	E
Rocky Mountain, South	Woodland Hardwoods	345	−0.78	−40.0	0.05	NE
South Central	Elm-Ash-Cottonwood	737	−1.68	−22.7	0.05	NE
South Central	Loblolly-Shortleaf Pine	3291	0.30	3.3	0.00	E
South Central	Minor types and nonstocked	842	−0.94	−11.6	0.03	E
South Central	Oak-Gum-Cypress	1264	−1.02	−8.1	0.02	E
South Central	Oak-Hickory	5806	−2.61	−32.3	0.03	NE
South Central	Oak-Pine	1093	−0.81	−10.2	0.02	E
South Central	Pinyon-Juniper	298	−0.30	−41.7	0.02	NE
South Central	Woodland Hardwoods	268	−0.28	−59.6	0.02	NE
Southeast	Elm-Ash-Cottonwood	173	−1.22	−12.7	0.07	E
Southeast	Loblolly-Shortleaf Pine	2667	0.39	4.1	0.01	E
Southeast	Longleaf-Slash Pine	1314	0.42	5.2	0.01	E
Southeast	Minor types and nonstocked	286	−0.68	−8.4	0.04	E
Southeast	Oak-Gum-Cypress	1311	−0.68	−5.0	0.01	E
Southeast	Oak-Hickory	3077	−1.34	−10.9	0.01	E
Southeast	Oak-Pine	982	−0.27	−2.5	0.01	E

^a^Equivalent (E) or not equivalent (NE)

**Table 2:** Estimates of belowground carbon stocks (Tg) and associated sampling errors (SE; %) for current US national greenhouse gas inventory (BGC_NGHGI_) [13] and adjusted estimates (BGC_ClimAdj_) by state

State	BGC_NGHGI_ (SE)	BGC_ClimAdj_ (SE)	Mean % difference
Alabama	176.23 (1.38)	198.13 (1.4)	12.4
Alaska^a^	234.3 (2.63)	252.84 (2.69)	7.9
Arizona	31.93 (4.27)	34.94 (4.2)	9.4
Arkansas	142.92 (1.61)	164.02 (1.59)	14.8
California	456.22 (1.37)	428.69 (1.27)	−6.0
Colorado	128.66 (1.92)	139.28 (1.9)	8.3
Connecticut	25.9 (3.51)	28.67 (3.5)	10.7
Delaware	4.66 (6.09)	5.13 (6.11)	10.1
Florida	105.88 (2.14)	111.18 (2.15)	5.0
Georgia	191.36 (1.48)	198.67 (1.48)	3.8
Idaho	209.16 (1.77)	212.81 (1.75)	1.7
Illinois	38.95 (2.86)	45.65 (2.86)	17.2
Indiana	45.67 (1.88)	53.27 (1.88)	16.6
Iowa	18.21 (4.21)	21.34 (4.22)	17.2
Kansas	14.7 (4.2)	17.26 (4.23)	17.4
Kentucky	109.67 (1.83)	140.42 (1.84)	28.0
Louisiana	133.93 (1.65)	145.01 (1.64)	8.3
Maine	127.35 (1.42)	140.69 (1.41)	10.5
Maryland	35.24 (3.31)	38.9 (3.31)	10.4
Massachusetts	43.72 (2.55)	47.78 (2.55)	9.3
Michigan	150.1 (1.38)	176.7 (1.39)	17.7
Minnesota	82.15 (1.35)	98.24 (1.34)	19.6
Mississippi	179.82 (1.3)	199.05 (1.32)	10.7
Missouri	103.99 (1.46)	121.17 (1.46)	16.5
Montana	197.84 (1.43)	202.28 (1.42)	2.2
Nebraska	6.64 (7.21)	7.65 (7.27)	15.2
Nevada	4.21 (13.01)	4.71 (12.85)	11.9
New Hampshire	62.93 (1.82)	69.28 (1.82)	10.1
New Jersey	23.15 (3.71)	25.57 (3.73)	10.5
New Mexico	45.86 (3.43)	50.12 (3.35)	9.3
New York	235.57 (1.05)	262.74 (1.05)	11.5
North Carolina	203.82 (1.23)	214.85 (1.24)	5.4
North Dakota	2.55 (11.19)	2.98 (11.26)	16.9
Ohio	93.07 (1.75)	108 (1.75)	16.0
Oklahoma	47.62 (2.54)	58.36 (2.54)	22.6
Oregon	478.52 (1.03)	427.63 (0.99)	−10.6
Pennsylvania	214 (1.1)	238.49 (1.1)	11.4
Rhode Island	4.96 (5.77)	5.44 (5.76)	9.7
South Carolina	109.52 (1.92)	112.13 (1.93)	2.4
South Dakota	7.86 (6.02)	8.68 (6.02)	10.4
Tennessee	160.47 (1.27)	203.39 (1.29)	26.7
Texas	129.47 (1.68)	149.23 (1.62)	15.3
Utah	34.85 (3.9)	38.62 (3.84)	10.8
Virginia	191.11 (1.18)	205.22 (1.19)	7.4
Washington	410.46 (1.29)	366.61 (1.26)	−10.7
West Virginia	169.05 (1.18)	188.03 (1.18)	11.2
Wisconsin	108.69 (1.14)	128.72 (1.13)	18.4
Wyoming	65.9 (5.54)	69.14 (5.49)	4.9
Total	5798.84	6167.71	6.4

^a^Coastal Alaska, only

Results from the random forests (RF) regressions indicated that aboveground biomass, stand age, and stand origin (i.e., planted versus natural regeneration) were useful predictors of climate-adjusted models of belowground carbon (BGC_ClimAdj_), as measured by their importance scores (Table 3). These variables accounted for 87 % of the total variation in belowground C stocks. Without employing aboveground biomass, 47 % of the total variability was accounted for, indicating that surrogates of climate (e.g., latitude and longitude) and knowledge of stand structure and management history (e.g., stand age and origin) may aid in understanding belowground C stocks.

**Table 3:** Summary of random forests model output and their importance scores (% IncMSE) for predicting refined estimates belowground C using basic stand structure, management, and physiographic variables

Variable	% IncMSE	Variable	% IncMSE
*With aboveground biomass*		*Without aboveground biomass*	
Aboveground biomass	34.5	Stand age	33.8
Stand age	20.7	Stand origin	29.1
Stand origin	19.0	Longitude	27.5
Latitude	16.8	Elevation	19.8
Longitude	15.9	Latitude	16.4
Elevation	13.6	Hopkins index	14.8
Hopkins index	12.8		
*R* ^2^	0.87	*R* ^2^	0.47
RMSE (Mg C ha-1)	3.72	RMSE (Mg C ha-1)	7.47

**Fig. 4:**
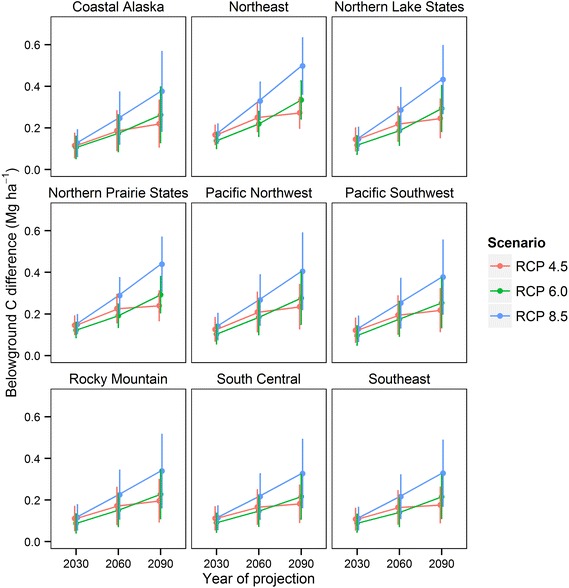
Regional differences in projected live-tree belowground C stocks compared to climate-sensitive estimates of belowground C derived from 30-year normal conditions (1960–1990). *Error bars* denote one standard deviation

The largest differences in projected live-tree belowground C stocks under future mean annual temperature (MAT) changes were positive, indicating decreases in belowground C stocks up to year 2090 (Fig. 4). Differences in projected belowground C stocks were similar across regions, with belowground stocks displaying approximately 0.1 Mg ha^−1^ less C in 2030 than assuming current normal climates. In 2090, differences comparing current versus future climates were highest in the US Northeast, representing 0.50 ± 0.13 Mg ha^−1^ less C (assuming an RCP8.5 scenario), or a reduction of approximately 3.4 % in belowground C stocks in the region.

## Discussion

Live tree belowground C estimated using allometric equations developed for individual trees and climate-specific predictions made at the forest stand level show markedly different patterns across various geographic regions and forest types in the US. Adjustments to the US’ approach to estimating live tree belowground C resulted in a C density estimate of 12 Mg ha^−1^ across the US and a 6.4 % increase in this forest C national stock when compared to current estimation strategies employed in the US NGHGI.

The largest differences between current and adjusted estimates of live tree belowground C were observed in the states of Oregon, Washington, and California, indicating climate-adjusted predictions estimated less belowground C compared to allometric-derived estimates in this region. Comparatively, this region contains the largest belowground C stocks in the US, quantified using model imputation strategies [18]. Similar differences but of a lesser magnitude were observed across the Coastal Plain region in the southeastern US. Such differences in these regions (i.e., the Pacific Northwest and Southeast) could point to the reliance on the allometric equations driven by aboveground metrics (i.e., tree diameter at breast height) in these carbon-dense stands. This may especially be the case for common tree species in the Pacific Northwest where the development of such allometric relationships is complicated by their relatively large above and belowground dimensions. In addition, intensive forest management regimes relying on artificial regeneration are common throughout these regions such that stand origin emerged as a useful predictor of live tree belowground C. Such a finding is analogous to recent work indicating greater partitioning to belowground components in managed plantations compared to natural-origin mature forests [9]. These patterns of partitioning may be reflective of the increased nutrient demands required to sustain the elevated levels of aboveground net primary production found in plantation systems [19], particularly on sites with lower nutrient capital such as in those found across the southeastern US. The importance of variables describing forest management regime and other forest conditions such as stand age in our random forests model output suggests that accounting for these aspects of forest origin and development is useful for understanding broad scale patterns in live tree belowground C.

The utilization of nationally consistent allometric equations [e.g., 12, 20] provides a general assessment of current C stocks. However, the appeal of employing BGC_ClimAdj_ estimates in the US NGHGI centers on utilizing standard forest inventory data collected in national forest inventories along with climate information. It is important to note that the model of Reich et al. [8] used here may be considered an empirical model. Such an empirical model may work well for data-rich countries with established national forest inventories. Other strategies, including process models, could potentially be employed to examine the carbon–climate relationship in forests. Incorporation of climate data enables evaluation of future climate change scenarios [e.g., 21] and their impacts on C monitoring efforts (e.g., United Nations Framework Convention on Climate Change submissions). As there are few studies that allow empirical observation of “true” coarse root C given the time and effort involved in destructively sampling belowground components, model validation procedures are limited for assessments at national scales. As an alternative, model-based approaches can be specified to be dynamic by incorporating future forest conditions and global change scenarios to determine their implications on C stocks and sequestration patterns. Such an approach using current climate conditions could provide general estimates of C stocks with associated uncertainty bounds for the temperate forests which occupy the US.

The majority of forest types displayed negative mean differences between current NGHGI and climate-adjusted models, indicating greater live tree belowground C stocks when using the adjusted models. The larger stocks in climate-adjusted models is partially a reflection of the ability of this framework to account for temperature-related shifts in patterns of belowground allocation within a species; a relationship held constant in current NGHGI models. In particular, a key component of the climate-adjusted model is an increased level of belowground allocation with decreasing temperatures given increasing nutrient limitation with colder temperatures [8]. These adjustments are reflected in the greater climate-adjusted estimates for northern latitude and high elevation forests (Fig. 2). Given the importance of climate variables in driving other belowground processes such as coarse root decomposition [22], the integration of these variables in models for describing other belowground stocks (e.g., dead roots) may not only aid in understanding C stock differences across large geographic scales but may also inform our understanding of other belowground processes.

The forecast of decreases in belowground C stocks up to year 2090 across all US regions and forest types is a function of projected increases in MAT and its role in the distribution of coarse root C stocks. National patterns in C stocks observed with US data indicated similar trends to the models presented in Reich et al. [8] showing that the proportion of total biomass to roots is greater in increasingly cold climates. Such assessments conducted in this analysis include a dynamic climate but assume constant aboveground biomass stocks, and a stand origin and phylogeny (e.g., conifer- or hardwood-dominated forest type). For example, the economic incentives of increasing C stocks to meet increased demand for wood for bioenergy [e.g., 23] has the potential to alter the proportion of planted compared to natural-origin stands at a national level. Although aridity was not found to influence the global distribution of coarse root biomass [8], what role might precipitation and/or its interaction with temperature and other climate variables play in determining future C sequestration patterns and stocks? Through designing national-scale models that incorporate climate parameters, the ability to quantify C stocks using relationships observed between climate and C dynamics is possible but is otherwise impracticable using allometric equations alone.

A particular concern from a physiological perspective relies in assessing carbon allocation tradeoffs in concert with changing climates. Changing allocation to belowground components can alter biomass accumulation and nutrient uptake [9], but without an assessment of additional carbon stocks from other pools (e.g., foliage and soil components), the role that future climate may play in determining overall stocks may be somewhat limited. Our approach in using current belowground C stocks from temperate forests across the US, with a range of current and projected climates, management histories, and stand structures, serves as a preliminary investigation of the role that climate may play on coarse root systems (e.g., Fig. 4). Carbon allocation to soil microbial biomass may be limited at northern sites with cooler climates [24] which would seemingly influence nutrient uptake and allocation within tree components. Process models [e.g., 25] may be well suited to examine such carbon allocation-climate tradeoffs, presenting a different approach compared to the empirical models examined here. In the interim, additional biomass data collected on all components from a range of species across sites with different climate regimes and management histories will aid in improving our understanding of carbon allocation patterns related to climate [26].

The finding that surrogates of climate (e.g., latitude and longitude) and knowledge of forest structure and management history (e.g., stand age and origin) were useful in predicting belowground C is encouraging when considering approaches to constructing a NGHGI. For example, any new strategy for estimating forest C pools in the US NGHGI requires “back casting” estimates to the 1990 reporting year [17]. Obtaining climate data from past years at large geographic scales may present less of a barrier to refinement of NGHGIs than other efforts (e.g., in situ field inventories in the 1990’s) to reduce the uncertainty associated with estimates of forest C and harvested wood products. Other nations could similarly adjust their C accounting practices using insight from this analysis to determine belowground stocks. Regardless of whether or not tree-level information from a national forest inventory is available, incorporating indicators of forest management history (e.g., proportion of planted versus natural origin stands) and variability in climate within a country (e.g., general trends in temperature), estimates of belowground C stocks could be developed that theoretically represent site and regional differences. In an era where destructive samples of belowground tree components is incomplete across the world’s forests, estimation strategies that merge the attributes of both allometric equations and C–climate relationships may refine forest C stocks estimations especially given emerging science that supports the relationships between climate, forest biomass, and C pools [1, 3, 8, 27].

## Conclusions

Numerous findings emerged from our investigation by incorporating climate variables into the estimation of belowground C stocks. First, climate variables can be used to adjust the US NGHGI estimates of belowground C stocks. Specifically, adjustment factors were specified to amend current coarse root C stocks estimated from allometric equations by incorporating mean annual temperature at various locations across the US. Second, for the US NGHGI, incorporating mean annual temperature increased national belowground C stocks by 6.4 %. In contrast, coarse root C stocks were projected to decrease through 2090, primarily due to lower partitioning to belowground components under warmer conditions. Third, whether or not a forest was planted or from natural origin, and its stand age were influential variables in determining belowground C stocks. Future work that integrates both climate and stand origin will increase our ability to predict belowground C stocks across regions containing a mixture of management and climate regimes. Finally, as a means of refining NGHGIs, climate-adjusted models depicting belowground C stocks should be adopted to incorporate the impacts of future global change and management scenarios on C sequestration patterns and stocks.

## Methods

### Study area

Forests across the US range are characterized by four major ecoclimatic zones, including polar, temperate humid, arid, and tropical humid types [28, 29]. The study area investigated here included forestlands across the contiguous US, spanning approximately 24° latitude (LAT) and 58° longitude (LONG) in addition to coastal Alaska (mean coordinates 57.87°N, 138.60°W). Mean annual temperature (MAT) ranged from −3.0 to 24.9 °C and precipitation (MAP) from 18 to 420 cm [30, 31]. Nine broad geographic regions were identified across the study area, ultimately containing 78 unique forest types [13] (Additional file 1: Table S1).

### Forest Inventory and Analysis data

The FIA program within the US Forest Service monitors forests by establishing permanent sample plots across the US in three phases [32]. During the inventory’s first phase, sample plot locations are established at an intensity of approximately 1 plot per 2400 ha. If the plot lies partially or wholly within a forested area, field personnel visit the site and establish a phase two (P2) inventory plot. Standard P2 inventory plots consist of four 7.32-m fixed radius subplots for a total plot area of approximately 0.07 ha where standing tree and site attributes are measured. Live trees with a DBH of at least 12.7 cm are measured on these subplots. Within each subplot a 2.07-m microplot is established where saplings with a DBH between 2.5 and 12.7 cm are measured.

All data were obtained from the publically-available FIA database (FIADB; [33]; http://apps.fs.fed.us/fiadb-downloads/datamart.html; download date 14 May 2014). If an FIA plot was remeasured at any point, only the most recent measurement was used in the analysis. Using the individual tree measurements, aboveground live-tree biomass (BIO_AG;_ Mg ha^−1^) was estimated by summing the bole, stump, top (excluding foliage), saplings, and woodland tree species (primarily those from dryland forests) components of each plot. Additional condition-level information including stand age (STDAGE; years) and a binary variable depicting stand origin (NAT; 1 = natural, 0 = artificial regeneration) were subsequently analyzed for each plot. As a bioclimatic measure, The Hopkins index ([34]; HI) standardizes the onset of spring for a given region and was computed for each FIA plot sampled relative to the mean LAT (40.35), LONG (−95.84), and elevation (ELEV; 2283 feet):

1 $$\begin{aligned} {\rm HI} = \left( {\frac{{\rm ELEV} - 2283}{100}} \right) + 4\,\left( {{\rm LAT} - 40.35} \right) + 1.25\,\left( { - 95.84 - {\rm LONG}} \right) \hfill \\ \ \hfill \\ \end{aligned}$$

The HI variable assumes that spring is delayed by one day for each 100-foot rise in ELEV, four days for each 1° increase in LAT, and 1.25 days for each 1° increase in LONG (e.g., more westward; [34]). In total, 70,126 FIA plots were analyzed for their belowground C.

### Belowground C in the US greenhouse gas inventory (BGC_NGHGI_)

The Intergovernmental Panel on Climate Change’s (IPCC) Good Practice Guidance considers forest C stocks associated with live aboveground, live belowground, dead wood, litter, and soil organic pools [35]. Hence, the focus of this analysis is on adjusting estimates of live belowground C, defined as all coarse living roots greater than 2 mm diameter [13]. Estimates of aboveground live C in the NGHGI are calculated using the component ratio method [36], but do not ultimately influence the analysis for belowground C here.

Belowground C for live trees is estimated in two stages using allometric equations. First, total aboveground biomass is estimated as a function of tree DBH [12]:

2 $${\rm Biomass} = \exp \left( {\alpha_{1,i} + \alpha_{2,i} \,\text{ln\,DBH} } \right)$$

where α_1,*i*_ and α_2,*i*_ are parameters for one of ten species groups [four hardwood groups (aspen/alder/cottonwood/willow, soft maple/birch, mixed hardwood, and hard maple/oak/hickory/beech), five conifer groups (cedar/larch, Douglas-fir, true fir/hemlock, pine, and spruce), and one woodland species group (juniper, oak, mesquite)]. Second, belowground root biomass is estimated as a ratio (BG_RATIO_) of root to total aboveground biomass [12]:

3 $${\rm BG}_{\rm RATIO} = \left\{ \begin{aligned} \exp \left( { - 1.6911 + \frac{0.8160}{\rm DBH}} \right),\,\,{\rm for}\,\,{\rm hardwood}\,\,{\rm species} \hfill \\ \exp \left( { - 1.5619 + \frac{0.6614}{\rm DBH}} \right),\,\,{\rm for}\,\,{\rm conifer}\,\,{\rm species} \hfill \\ \end{aligned} \right.$$

Hence, parameters indicate BG_RATIO_ will decrease for larger DBH trees and that for a fixed DBH, BG_RATIO_ will be larger for conifer compared to hardwood species. Belowground biomass was estimated by multiplying the values obtained from Eqs. 2 and 3, then converted to C by multiplying by 0.5, assuming 50 % of biomass is C [35]. Estimates of belowground C were scaled to the plot level and are hereby abbreviated as BGC_NGHGI_.

### Belowground C from climate-derived models (BGC_Clim_)

Recent investigations of the global distribution of biomass within forests have provided insight for comparing size- and species-specific predictions (i.e., allometric equations and proportional ratios) with climate-sensitive estimations of belowground C [e.g., 8]. Using the relationship observed between mean annual temperature and root mass fraction, the model used to determine a climate-derived estimate of belowground C was parameterized with global data compiled from various sources including Usoltsev [37], Luo et al. [38], Cannell [39], and from over 1000 additional forest stands including the US [8]. A total of 3043 of these stands contained measurements of belowground biomass. The motivation for the development of this model was to assess the distribution of biomass in roots along a temperature and precipitation spectrum [8]. We estimated a climate-sensitive prediction of belowground biomass (BGB_Clim_) using the model of Reich et al. [8] (Table 4). In addition to MAT and NAT, a dummy variable indicating whether or not the FIA plot was primarily dominated by hardwoods or conifers (HDWD) and stem biomass (BIO_STEM_; Mg ha^−1^) of live trees were used to estimate BGB_Clim_. Thirty-year (1961–1990) climate data (i.e., MAT) were obtained by specifying LAT, LONG, and ELEV of each FIA plot location to a spline surface model developed from climate station data across forests of North America [30, 31]. We assigned the HDWD variable using the FIA forest type code [40] by separating conifer-dominated forest type codes (i.e., FORTYPCD ≤ 409) with hardwood-dominated codes (FORTYPCD ≥ 500). By incorporating measures of aboveground biomass, these allometric relationships allow one to capture the variability observed across a range of stand structures and ages. Continuous independent variables were centered prior to applying the Reich et al. [8] model to the FIA plots. When standardized for a given MAT and aboveground stem biomass, the model of Reich et al. [8] indicates conifer forests tended to have a smaller root mass fraction than hardwood forests. Values for BGB_Clim_ were converted to BGC_Clim_ by multiplying by 0.5 [35].

**Table 4:** Model parameters and model form Reich et al. [8] used in this analysis for estimating climate-adjusted belowground biomass (BGB_Clim_)

Term	Parameter	Value	SE
b_0_	Intercept	−0.18088	0.021062
b_1_	HDWD	0.0172682	0.005315
b_2_	NAT	0.0018117	0.005256
b_3_	MAT	−0.003032	0.000564
b_4_	log(BIO_STEM_)	0.7940911	0.011187
b_5_	HDWD × NAT	−0.000591	0.005291
b_6_	HDWD × (MAT − 9.1374)	−0.001423	0.000566
b_7_	HDWD × (log(BIO_STEM_ − 1.88807))	−0.031736	0.008798
b_8_	NAT × (MAT − 9.1374)	−0.000555	0.000574
b_9_	NAT × (log(BIO_STEM_ − 1.88807))	0.021458	0.010028
b_10_	(MAT − 9.1374) × (log(BIO_STEM_ − 1.88807))	0.0020443	0.001155
b_11_	HDWD × NAT × (MAT − 9.1374)	0.0020594	0.000566
b_12_	HDWD × NAT × (log(BIO_STEM_ − 1.88807))	0.0269889	0.010957
b_13_	HDWD × (MAT − 9.1374) × (log(BIO_STEM_ − 1.88807))	0.0050601	0.001197
b_14_	NAT × (MAT − 9.1374) × (log(BIO_STEM_ − 1.88807))	0.0016555	0.001206

Parameters are: a binary variable depicting general forest type (HDWD; 1 = hardwood-dominated, 0 = conifer-dominated), stem biomass of live trees (BIO_STEM_; Mg ha^−1^), a binary variable depicting stand origin (NAT; 1 = natural, 0 = artificial regeneration), mean annual temperature (MAT; °C), and log is to the base 10

In $$\begin{aligned} \,\log (\rm BGBClim) &= b0 + b1\left( {\rm HDWD} \right) + b2\left( {\rm NAT} \right) + b3\left( {\rm MAT} \right) + b4\left( {\log \left( {\rm BIOSTEM} \right)} \right) + \,b5\left( {\left( {{\rm HDWD} \times {\rm NAT}} \right)} \right) + \hfill \\ & \quad b6\left( {\left( {{\rm HDWD} \times \left( {{\rm MAT} - 9.1374} \right)} \right)} \right) + \,b7\left( {\left( {{\rm HDWD} \times \left( {\log \left( {\rm BIOSTEM} \right) - 1.88807} \right)} \right)} \right) + \hfill \\ & \quad b8\left( {\left( {{\rm NAT} \times \left( {{\rm MAT} - 9.1374} \right)} \right)} \right) + \,b9\left( {\left( {{\rm NAT} \times \left( {\log \left( {\rm BIOSTEM} \right) - 1.88807} \right)} \right)} \right) + \hfill \\ & \quad b10\left( {\left( {\left( {{\rm MAT} - 9.1374} \right) \times \left( {\log \left( {\rm BIOSTEM} \right) - 1.88807} \right)} \right)} \right) + \hfill \\ & \quad b11\left( {\left( {\rm HDWD} \right) \times \left( {\rm NAT} \right) \times \left( {{\rm MAT} - 8.8508} \right)} \right) + \hfill \\ & \quad b12\left( {\left( {\rm HDWD} \right) \times \left( {\rm NAT} \right) \times \left( {\log \left( {\rm BIOSTEM} \right) - 1.88807} \right)} \right) + \hfill \\ & \quad b13\left( {\left( {\rm HDWD} \right) \times \left( {{\rm MAT} - 8.8508} \right) \times \left( {\log \left( {\rm BIOSTEM} \right) - 1.88807} \right)} \right) + \hfill \\ & \quad b14\left( {\left( {\rm NAT} \right) \times \left( {{\rm MAT} - 8.8508} \right) \times \left( {\log \left( {\rm BIOSTEM} \right) - 1.88807} \right)} \right) \hfill \\ \end{aligned}$$

### Climate-adjusted models of belowground C (BGC_ClimAdj_)

Current models of belowground C in the NGHGI (i.e., BGC_NGHGI_) could likely be adjusted by incorporating climate-derived estimates of belowground C (i.e., BGC_Clim_). From a NGHGI reporting perspective, estimates of BGC would need to be made on individual plots, then “back cast” to contemporary estimates using the 1990 baseline reporting year [17]. Adjustment factors were estimated to align allometric- and climate-derived estimates:

4 $${\text{AdjFactor}} = \frac{{\text{BGC}}_{{\rm Clim}}}{{\text{BGC}}_{{\rm NGHGI}}}$$

where AdjFactor is the ratio of climate- to allometric-derived belowground C for a specific forest type found in a given geographic region. New climate-adjusted estimates of belowground C (BGC_ClimAdj_) are then:

5 $${\text{BGC}}_{\text{ClimAdj}} = {\text{BGC}}_{\text{NGHGI}} \times {\text{AdjFactor}}$$

where BGC_ClimAdj_ was computed for each FIA plot record.

### Analyzing belowground C model differences

We conducted equivalence tests comparing BGC_NGHGI_ and BGC_ClimAdj_ model predictions using two one-sided tests [41]. Equivalence tests are commonly applied in the forest science literature and are advantageous in that they can be used in model validation by assuming a null hypothesis of dissimilarity [42]. Equivalence tests are unlike statistical goodness-of-fit approaches and instead examine dissimilarity. Dissimilarity in the equivalence test was specified using a threshold of ±25 %. This threshold allows for a moderate amount of disagreement between the various model predictions, with non-equivalence suggesting biological disparities in C stocks. Differences between BGC_NGHGI_ and BGC_ClimAdj_ models were mapped across the US to examine geographic trends in estimates of belowground C when using each approach. We computed US state-level population estimates of belowground C (Tg) using BGC_NGHGI_ and BGC_ClimAdj_ models and compared mean percent differences for the two estimation strategies.

Nonparametric random forests (RF; [43]) were implemented in R [44] to identify variables that were effective in describing BGC_ClimAdj_. Recognizing that not all users may implement climate data in determining forest C stocks, seven variables from the FIADB (BIO_AG_, STDAGE, NAT, LAT, LONG, ELEV, and HI) were chosen for incorporation into the RF. This method involved building a set of regression trees based on bootstrapped samples of the belowground C data. We similarly fit a RF model without BIO_AG_ to examine how belowground C can be predicted without knowledge of aboveground biomass stocks.

### Belowground C in future scenarios

Current CMIP5 models [45] as described in the fifth assessment report (AR5) of the IPCC [46] were obtained using three scenarios (RCP 4.5, RCP 6.0, RCP 8.5; [30]). An ensemble of 17 AR5 model predictions was used for each RCP scenario. Provided that differences in belowground C could exist in future global change scenarios (i.e., changes in MAT at various locations in the US), climate data were obtained for the 30-year normal (1961–1990) and years 2030, 2060, and 2090. To gain insight into temperature-related patterns and their influence on belowground C, climate-sensitive estimates were made using these new MAT values in future years (while holding fixed the variables HDWD, NAT, and BIO_STEM_) to assess the variation in belowground C assuming future scenarios. While BIO_STEM_ will fluctuate in response to trends in forest growth and mortality, this simulation was specifically designed to understand the influence of MAT in future belowground C stocks.

## Additional file

Additional file 1: Table S1. Summary of live-tree belowground C estimates (Mg ha^−1^) from the current US national greenhouse gas inventory (BGC_NGHGI_; Smith et al. 2013), climate-derived predictions (BGC_Clim_; Reich et al. 2014), and adjustment factors (AdjFactor) used to amend BGC_NGHGI_ estimates by taking into account climate-derived predictions.

## Abbreviations

C: carbon
US NGHGI: United States’ National Greenhouse Gas Inventory
GHG: greenhouse gas
FIA: Forest Inventory and Analysis
LAT: latitude
LONG: longitude
MAT: mean annual temperature
MAP: mean annual precipitation
BIO_AG_: aboveground live-tree biomass
STDAGE: stand age
NAT: natural regeneration
RF: random forests
HI: Hopkins index
ELEV: elevation
BGC_NGHGI_: belowground carbon in the US greenhouse gas inventory
IPCC: Intergovernmental Panel on Climate Change
DBH: tree diameter at breast height
BG_RATIO_: belowground root biomass ratio
BGC_Clim_: belowground carbon from climate-derived models
BGB_Clim_: belowground biomass from climate-derived models
HDWD: hardwood forest type
BIO_STEM_: stem biomass
FORTYPCD: forest type code
BGC_ClimAdj_: climate-adjusted models of belowground carbon
AdjFactor: the ratio of climate- to allometric-derived belowground carbon
