# Supplementary material for: Comparisons of allometric and climate-derived estimates of tree coarse root carbon stocks in forests of the United States
Source: Carbon Balance Manag. 2015 Sep 4;10:20. doi: 10.1186/s13021-015-0032-7 (PMC4559578; doi:10.1186/s13021-015-0032-7)
Supplement: Additional file 1: Table S1. — Summary of live-tree belowground C estimates (Mg ha−1) from the current US national greenhouse gas inventory (BGCNGHGI; Smith et al. 2013), climate-derived predictions (BGCClim; Reich et al. 2014), and adjustment factors (AdjFactor) used to amend BGCNGHGI estimates by taking into account climate-derived predictions. [file 13021_2015_32_MOESM1_ESM.docx]

**Table S1.** Summary of live-tree belowground C estimates (Mg ha^-1^) from the current US national greenhouse gas inventory (BGC_NGHGI_; Smith et al. 2013), climate-derived predictions (BGC_Clim_; Reich et al. 2014), and adjustment factors (AdjFactor) used to amend BGC_NGHGI_ estimates by taking into account climate-derived predictions.

| **Region** | **Forest type** | ***n*** | **Mean** | **SE** | **Min** | **Max** | **Mean** | **SE** | **Min** | **Max** | **AdjFactor** |
| --- | --- | --- | --- | --- | --- | --- | --- | --- | --- | --- | --- |
|  |  | | BGC_NGHGI_ | | | | BGC_Clim_ | | | |  |
| All regions | All forest types | 70126 | 11.60 | 0.04 | 0.00 | 353.63 | 12.20 | 0.04 | 0.00 | 272.29 | 1.05 |
|  |  |  |  |  |  |  |  |  |  |  |  |
| Coastal Alaska | Aspen-Birch | 78 | 5.02 | 0.48 | 0.05 | 19.10 | 6.73 | 0.65 | 0.07 | 25.59 | 1.34 |
| Coastal Alaska | Fir-Spruce-Mt. Hemlock | 342 | 13.54 | 0.60 | 0.03 | 57.57 | 13.14 | 0.58 | 0.03 | 55.84 | 0.97 |
| Coastal Alaska | Hemlock-Sitka Spruce | 571 | 25.00 | 0.68 | 0.02 | 96.63 | 22.25 | 0.60 | 0.01 | 86.00 | 0.89 |
| Coastal Alaska | Minor Types and Nonstocked | 85 | 5.52 | 0.94 | 0.01 | 62.87 | 6.29 | 1.07 | 0.01 | 71.67 | 1.14 |
| Coastal Alaska | Spruce-Fir | 105 | 2.61 | 0.22 | 0.02 | 10.35 | 3.21 | 0.27 | 0.03 | 12.73 | 1.23 |
| Northeast | Aspen-Birch | 367 | 8.79 | 0.25 | 0.02 | 23.30 | 10.38 | 0.30 | 0.02 | 27.49 | 1.18 |
| Northeast | Elm-Ash-Cottonwood | 205 | 10.36 | 0.44 | 0.05 | 27.86 | 12.12 | 0.52 | 0.05 | 32.60 | 1.17 |
| Northeast | Maple-Beech-Birch | 3845 | 13.71 | 0.10 | 0.02 | 38.15 | 15.50 | 0.11 | 0.03 | 43.10 | 1.13 |
| Northeast | Minor Types and Nonstocked | 346 | 10.78 | 0.35 | 0.04 | 33.75 | 11.75 | 0.39 | 0.04 | 36.79 | 1.09 |
| Northeast | Oak-Hickory | 2509 | 15.19 | 0.13 | 0.02 | 43.96 | 16.87 | 0.14 | 0.03 | 48.79 | 1.11 |
| Northeast | Oak-Pine | 247 | 13.53 | 0.39 | 0.14 | 29.40 | 14.21 | 0.41 | 0.14 | 30.87 | 1.05 |
| Northeast | Spruce-Fir | 900 | 8.43 | 0.15 | 0.04 | 28.38 | 9.02 | 0.16 | 0.04 | 30.36 | 1.07 |
| Northeast | White-Red-Jack Pine | 380 | 15.58 | 0.31 | 0.29 | 34.08 | 15.58 | 0.31 | 0.29 | 34.08 | 1.00 |
| Northern Lake States | Aspen-Birch | 2477 | 6.28 | 0.08 | 0.01 | 38.09 | 8.10 | 0.11 | 0.02 | 49.14 | 1.29 |
| Northern Lake States | Elm-Ash-Cottonwood | 971 | 8.32 | 0.17 | 0.03 | 31.03 | 10.24 | 0.20 | 0.04 | 38.16 | 1.23 |
| Northern Lake States | Maple-Beech-Birch | 2956 | 11.57 | 0.09 | 0.02 | 33.94 | 13.66 | 0.11 | 0.02 | 40.05 | 1.18 |
| Northern Lake States | Minor Types and Nonstocked | 460 | 6.25 | 0.26 | 0.01 | 27.46 | 7.19 | 0.29 | 0.01 | 31.58 | 1.15 |
| Northern Lake States | Oak-Hickory | 1845 | 10.24 | 0.13 | 0.04 | 35.21 | 12.29 | 0.15 | 0.05 | 42.25 | 1.20 |
| Northern Lake States | Spruce-Fir | 1854 | 6.42 | 0.10 | 0.02 | 26.16 | 6.93 | 0.11 | 0.02 | 28.26 | 1.08 |
| Northern Lake States | White-Red-Jack Pine | 935 | 8.70 | 0.19 | 0.02 | 47.76 | 9.22 | 0.20 | 0.02 | 50.63 | 1.06 |
| Northern Prairie States | Elm-Ash-Cottonwood | 342 | 9.88 | 0.31 | 0.14 | 31.11 | 11.76 | 0.36 | 0.16 | 37.02 | 1.19 |
| Northern Prairie States | Minor Types and Nonstocked | 465 | 9.72 | 0.29 | 0.03 | 31.20 | 10.98 | 0.33 | 0.03 | 35.25 | 1.13 |
| Northern Prairie States | Oak-Hickory | 3265 | 10.58 | 0.09 | 0.02 | 35.87 | 12.38 | 0.10 | 0.03 | 41.97 | 1.17 |
| Northern Prairie States | Oak-Pine | 207 | 8.40 | 0.28 | 0.52 | 24.78 | 9.15 | 0.31 | 0.56 | 27.01 | 1.09 |
| Northern Prairie States | Ponderosa Pine | 164 | 6.71 | 0.36 | 0.04 | 25.07 | 7.25 | 0.39 | 0.04 | 27.07 | 1.08 |
| Pacific Northwest, Eastside | Douglas-fir | 992 | 13.51 | 0.32 | 0.01 | 65.01 | 13.24 | 0.31 | 0.01 | 63.71 | 0.98 |
| Pacific Northwest, Eastside | Fir-Spruce-Mt. Hemlock | 948 | 16.40 | 0.37 | 0.02 | 63.75 | 15.58 | 0.36 | 0.02 | 60.56 | 0.95 |
| Pacific Northwest, Eastside | Lodgepole Pine | 571 | 7.42 | 0.25 | 0.03 | 38.58 | 7.79 | 0.26 | 0.03 | 40.51 | 1.05 |
| Pacific Northwest, Eastside | Minor Types and Nonstocked | 213 | 2.75 | 0.28 | 0.01 | 27.23 | 3.66 | 0.37 | 0.02 | 36.22 | 1.33 |
| Pacific Northwest, Eastside | Other Western Softwoods | 505 | 5.73 | 0.48 | 0.01 | 68.23 | 5.78 | 0.48 | 0.01 | 68.92 | 1.01 |
| Pacific Northwest, Eastside | Ponderosa Pine | 1446 | 8.95 | 0.16 | 0.02 | 39.95 | 9.31 | 0.17 | 0.02 | 41.55 | 1.04 |
| Pacific Northwest, Eastside | Western Larch | 136 | 16.35 | 0.85 | 0.16 | 57.50 | 15.69 | 0.82 | 0.15 | 55.20 | 0.96 |
| Pacific Northwest, Westside | Alder-Maple | 226 | 17.83 | 0.88 | 0.17 | 65.85 | 17.66 | 0.87 | 0.16 | 65.19 | 0.99 |
| Pacific Northwest, Westside | Douglas-fir | 2130 | 36.22 | 0.55 | 0.02 | 150.69 | 30.42 | 0.46 | 0.01 | 126.58 | 0.84 |
| Pacific Northwest, Westside | Fir-Spruce-Mt. Hemlock | 553 | 30.47 | 0.91 | 0.02 | 96.47 | 26.20 | 0.79 | 0.01 | 82.97 | 0.86 |
| Pacific Northwest, Westside | Hemlock-Sitka Spruce | 552 | 40.76 | 0.96 | 0.01 | 142.31 | 33.83 | 0.80 | 0.01 | 118.12 | 0.83 |
| Pacific Northwest, Westside | Minor Types and Nonstocked | 355 | 14.18 | 0.75 | 0.01 | 77.14 | 13.89 | 0.73 | 0.01 | 75.60 | 0.98 |
| Pacific Southwest | California Mixed Conifer | 946 | 23.00 | 0.53 | 0.01 | 86.99 | 20.70 | 0.48 | 0.01 | 78.29 | 0.90 |
| Pacific Southwest | Douglas-fir | 139 | 31.82 | 1.90 | 1.05 | 119.72 | 27.37 | 1.63 | 0.90 | 102.96 | 0.86 |
| Pacific Southwest | Fir-Spruce-Mt. Hemlock | 243 | 25.52 | 1.09 | 0.24 | 88.46 | 22.71 | 0.97 | 0.21 | 78.73 | 0.89 |
| Pacific Southwest | Minor Types and Nonstocked | 116 | 8.32 | 1.19 | 0.01 | 57.61 | 8.56 | 1.23 | 0.01 | 59.34 | 1.03 |
| Pacific Southwest | Other Western Softwoods | 382 | 8.00 | 0.46 | 0.02 | 50.17 | 8.16 | 0.47 | 0.02 | 51.18 | 1.02 |
| Pacific Southwest | Pinyon-Juniper | 31 | 2.10 | 0.44 | 0.05 | 8.02 | 2.67 | 0.56 | 0.06 | 10.19 | 1.27 |
| Pacific Southwest | Ponderosa Pine | 242 | 10.19 | 0.57 | 0.02 | 60.91 | 10.19 | 0.57 | 0.02 | 60.91 | 1.00 |
| Pacific Southwest | Redwood | 64 | 59.27 | 7.06 | 0.87 | 353.63 | 45.64 | 5.44 | 0.67 | 272.29 | 0.77 |
| Pacific Southwest | Tanoak-Laurel | 207 | 25.10 | 1.14 | 0.35 | 95.29 | 23.59 | 1.07 | 0.33 | 89.57 | 0.94 |
| Pacific Southwest | Western Oak | 778 | 9.79 | 0.33 | 0.05 | 65.43 | 10.77 | 0.37 | 0.06 | 71.97 | 1.10 |
| Rocky Mountain, North | Aspen-Birch | 98 | 4.43 | 0.44 | 0.03 | 22.98 | 5.90 | 0.58 | 0.04 | 30.56 | 1.33 |
| Rocky Mountain, North | Douglas-fir | 1659 | 10.62 | 0.2 | 0.02 | 56.42 | 10.73 | 0.21 | 0.02 | 56.99 | 1.01 |
| Rocky Mountain, North | Fir-Spruce-Mt. Hemlock | 1467 | 11.97 | 0.24 | 0.04 | 61.47 | 11.97 | 0.24 | 0.04 | 61.47 | 1.00 |
| Rocky Mountain, North | Lodgepole Pine | 828 | 9.91 | 0.22 | 0.01 | 32.62 | 10.20 | 0.23 | 0.01 | 33.59 | 1.03 |
| Rocky Mountain, North | Minor Types and Nonstocked | 331 | 1.53 | 0.14 | 0.00 | 23.35 | 2.23 | 0.20 | 0.01 | 34.09 | 1.46 |
| Rocky Mountain, North | Other Western Softwoods | 329 | 11.29 | 0.64 | 0.04 | 52.59 | 11.07 | 0.62 | 0.04 | 51.53 | 0.98 |
| Rocky Mountain, North | Ponderosa Pine | 499 | 6.66 | 0.29 | 0.01 | 41.58 | 7.13 | 0.31 | 0.01 | 44.49 | 1.07 |
| Rocky Mountain, North | Western Larch | 128 | 12.86 | 0.79 | 0.10 | 42.07 | 12.60 | 0.77 | 0.10 | 41.23 | 0.98 |
| Rocky Mountain, South | Aspen-Birch | 647 | 8.03 | 0.23 | 0.10 | 33.66 | 9.79 | 0.29 | 0.12 | 41.07 | 1.22 |
| Rocky Mountain, South | Douglas-fir | 420 | 10.20 | 0.33 | 0.27 | 43.50 | 10.61 | 0.34 | 0.28 | 45.24 | 1.04 |
| Rocky Mountain, South | Fir-Spruce-Mt. Hemlock | 1061 | 11.38 | 0.22 | 0.03 | 45.47 | 11.72 | 0.23 | 0.03 | 46.83 | 1.03 |
| Rocky Mountain, South | Lodgepole Pine | 359 | 10.21 | 0.31 | 0.05 | 36.26 | 10.61 | 0.32 | 0.05 | 37.71 | 1.04 |
| Rocky Mountain, South | Minor Types and Nonstocked | 166 | 4.49 | 0.40 | 0.02 | 31.23 | 5.26 | 0.47 | 0.02 | 36.54 | 1.17 |
| Rocky Mountain, South | Pinyon-Juniper | 647 | 1.42 | 0.06 | 0.01 | 13.82 | 1.83 | 0.08 | 0.01 | 17.83 | 1.29 |
| Rocky Mountain, South | Ponderosa Pine | 924 | 7.52 | 0.16 | 0.08 | 41.52 | 7.97 | 0.17 | 0.09 | 44.01 | 1.06 |
| Rocky Mountain, South | Woodland Hardwoods | 345 | 1.95 | 0.13 | 0.01 | 22.53 | 2.72 | 0.18 | 0.01 | 31.54 | 1.40 |
| South Central | Elm-Ash-Cottonwood | 737 | 7.32 | 0.23 | 0.00 | 38.97 | 9.00 | 0.28 | 0.00 | 47.93 | 1.23 |
| South Central | Loblolly-Shortleaf Pine | 3291 | 10.03 | 0.12 | 0.00 | 57.54 | 9.73 | 0.11 | 0.00 | 55.82 | 0.97 |
| South Central | Minor Types and Nonstocked | 842 | 7.85 | 0.24 | 0.00 | 33.48 | 8.79 | 0.27 | 0.00 | 37.50 | 1.12 |
| South Central | Oak-Gum-Cypress | 1264 | 12.77 | 0.24 | 0.00 | 72.68 | 13.80 | 0.26 | 0.00 | 78.49 | 1.08 |
| South Central | Oak-Hickory | 5806 | 8.17 | 0.09 | 0.00 | 48.2 | 10.78 | 0.12 | 0.00 | 63.63 | 1.32 |
| South Central | Oak-Pine | 1093 | 8.12 | 0.19 | 0.00 | 40.42 | 8.93 | 0.21 | 0.00 | 44.46 | 1.10 |
| South Central | Pinyon-Juniper | 298 | 0.72 | 0.04 | 0.01 | 4.42 | 1.02 | 0.06 | 0.02 | 6.28 | 1.42 |
| South Central | Woodland Hardwoods | 268 | 0.47 | 0.03 | 0.01 | 2.86 | 0.75 | 0.05 | 0.01 | 4.57 | 1.60 |
| Southeast | Elm-Ash-Cottonwood | 173 | 9.36 | 0.57 | 0.02 | 32.66 | 10.58 | 0.65 | 0.03 | 36.91 | 1.13 |
| Southeast | Loblolly-Shortleaf Pine | 2667 | 9.83 | 0.13 | 0.00 | 41.53 | 9.44 | 0.12 | 0.00 | 39.87 | 0.96 |
| Southeast | Longleaf-Slash Pine | 1314 | 8.47 | 0.17 | 0.02 | 44.27 | 8.05 | 0.17 | 0.02 | 42.05 | 0.95 |
| Southeast | Minor Types and Nonstocked | 286 | 8.44 | 0.46 | 0.02 | 35.50 | 9.12 | 0.49 | 0.02 | 38.33 | 1.08 |
| Southeast | Oak-Gum-Cypress | 1311 | 13.51 | 0.26 | 0.02 | 52.11 | 14.19 | 0.27 | 0.02 | 54.72 | 1.05 |
| Southeast | Oak-Hickory | 3077 | 12.15 | 0.13 | 0.00 | 44.11 | 13.49 | 0.15 | 0.00 | 48.96 | 1.11 |
| Southeast | Oak-Pine | 982 | 9.10 | 0.22 | 0.02 | 31.41 | 9.37 | 0.22 | 0.02 | 32.35 | 1.03 |
